# Association between plasma short-chain fatty acids and inflammation in human immunodeficiency virus-associated neurocognitive disorder: a pilot study

**DOI:** 10.1186/s12944-025-02477-x

**Published:** 2025-02-21

**Authors:** Xue Chen, Jiaqi Wei, Ling Zhang, Hu Wang, Yang Zhang, Zhen Li, Xia Wang, Lifeng Liu, Yulin Zhang, Tong Zhang

**Affiliations:** 1 Clinical and Research Center for Infectious Diseases, Beijing Youan Hospital, Capital Medical University, Beijing, China; 2 Beijing Key Laboratory for HIV/AIDS Research, Beijing Youan Hospital, Capital Medical University, Beijing, China; 3 Beijing Youan Hospital, Beijing Institute of Hepatology, Capital Medical University, Beijing, China; 4 Department of Respiratory and Critical Care Medicine, Beijing Youan Hospital, Capital Medical University, Beijing, China

**Keywords:** Short-chain fatty acids, Gut microbiota, HIV-associated neurocognitive disorder, Inflammation, Predictive biomarkers

## Abstract

**Background and aims:**

Short-chain fatty acids (SCFAs), key metabolites produced by gut microbiota, have neuroprotective effects in neurodegenerative diseases by modulating immune responses. However, their role in human immunodeficiency virus (HIV)-associated neurocognitive disorder (HAND) remains largely unexplored.

**Methods:**

We recruited HAND patients, HIV Control, and healthy controls (HC). Plasma SCFAs and SCFA-producing gut microbiota were quantified via gas chromatography-mass spectrometry and fecal metagenomic analysis. Inflammatory cytokine levels were measured using liquid chromatography. Receiver operating characteristic (ROC) curves were generated to evaluate the predictive accuracy of SCFAs for HAND.

**Results:**

Plasma SCFAs were significantly reduced in HAND patients, correlating with a decrease in SCFA-producing gut bacteria, such as *Prevotella* and its related species. Reduced SCFAs were positively correlated with pro-inflammatory cytokines and cognitive impairment, while being negatively correlated with anti-inflammatory cytokines. ROC curve analysis demonstrated that several SCFAs exhibited strong predictive accuracy for HAND status.

**Conclusions:**

SCFAs may influence cognitive function by modulating inflammatory responses, and identifies plasma SCFAs as potential biomarkers and therapeutic targets for HAND. Further investigation is needed to delineate the mechanisms that SCFAs influence HAND pathology.

**Supplementary Information:**

The online version contains supplementary material available at 10.1186/s12944-025-02477-x.

## Introduction

Growing evidence suggests that the microbiota-gut-brain axis is associated with various neurocognitive disorders, including Alzheimer’s disease (AD) and Parkinson’s disease (PD) [1, 2]. This axis forms a complex and dynamic bidirectional communication network, linking the gut and the brain through both direct and indirect pathways [3–5]. Notably, gut microbiota-derived metabolites serve as key regulators of brain function, regulating peripheral and local signaling via immunological, neuronal, and endocrinological pathways [6]. Human immunodeficiency virus (HIV)-associated neurocognitive disorder (HAND), like other neurocognitive disorders, is influenced by the microbiota and its metabolites [7]. Our previous study characterized the gut microbiota and plasma metabolomics in HAND patients, highlighting the potential role of gut microbiota-derived metabolites in the pathogenesis of HAND [8]. However, the crosstalk between HAND and the microbiota-gut-brain axis remains to be fully elucidated.

Short-chain fatty acids (SCFAs) are considered key mediators in microbiota-gut-brain crosstalk [9], exerting significant effects on central nervous system (CNS) diseases by regulating inflammatory responses, neuronal apoptosis, oxidative stress, and maintaining the integrity of the blood-brain barrier (BBB) [9, 10]. Notably, SCFAs like acetate, propionate, and butyrate are strongly linked to anti-inflammatory effects within the CNS. For example, butyrate has been shown to reduce inflammatory responses in primary, brain-derived microglial cells exposed to lipopolysaccharide (LPS) [11]. Multiple studies emphasize the immunomodulatory role of acetate in CNS disorders by reducing pro-inflammatory cytokine levels, suppressing microglial activation toward a pro-inflammatory phenotype, inhibiting astrocyte activation, and attenuating adenosine-mediated inflammatory responses [12–14]. Intriguingly, fecal SCFAs levels were notably lower in individuals with PD compared to healthy controls, whereas plasma SCFAs levels showed a paradoxical increase in PD and were found to correlate with disease severity [15]. Acetic acid and propanoic acid could potentially serve as biomarkers for distinguishing PD from healthy states [16, 17]. In individuals with AD, lower serum acetate concentrations have been observed, which correlate with an increased risk of AD [18]. Another study indicated a progressive decrease in SCFAs levels in fecal samples from AD patients [19]. Acetate exerts neuroprotective effects against neuroinflammation by upregulating GPR41 and inhibiting the ERK/JNK/NF-κB signaling pathway, underscoring its potential in AD prevention [14]. Long-term dietary SCFA supplementation during the early stages of aging can regulate neuroenergetics to alleviate AD [20].

Neuroinflammation is a hallmark feature of HAND, and in other neurological disorders, previous studies have shown that SCFAs, metabolites derived from gut microbiota, can influence neurocognitive function by modulating neuroinflammation. However, the association between SCFAs, inflammatory cytokines, and HAND severity remains unclear. In our study, we measured the concentration of plasma SCFAs in patients with HAND using gas chromatography-mass spectrometry (GC-MS) and conducted fecal metagenomic analysis to assess changes in SCFA-producing gut microbiota. Additionally, we employed liquid chromatography chips to assess inflammatory factor expression in HAND patients, correlating these findings with SCFAs levels. Our objective was to investigate the relationship between plasma SCFAs and inflammation in HAND, and explore the therapeutic potential of targeting SCFAs for HAND.

## Methods

### Participants recruitment

Participants were enrolled in our study from Beijing Youan Hospital between February and July 2023. All participants were divided into three groups: the HAND group, the HIV control group, and the healthy control (HC) group.

Inclusion criteria included an age range of 18–65 years, the ability to independently complete neurocognitive tests, and a confirmed HIV diagnosis for HAND and HIV control groups. Exclusion criteria included other neurocognitive disorders (e.g., Alzheimer’s disease, Parkinson’s disease, frontotemporal dementia, vascular disease, and frontotemporal lobar degeneration), other mental illnesses (e.g., depressive disorder and bipolar disorder), gastrointestinal conditions (e.g., irritable bowel syndrome, inflammatory bowel disease and colon cancer), other infectious diseases (e.g., hepatitis B virus and hepatitis C virus infection), the use of antibiotics, probiotics, or acid-suppressing drugs within the past three months, and unusual dietary habits (including restrictive eating patterns, extreme or fad diets, food pairing and combinations, non-normal eating behaviors, nutritional imbalances, psychological or medical influences).

The study was approved by the Beijing Youan hospital’s ethics committee (JYKL 2023–057), and all participants provided signed informed consent.

### Cognitive function Assessment

Montreal cognitive assessment (MoCA), a general screening tool, used to assess cognitive function across various domains, including attention, memory, language, and executive functions, through tasks such as drawing a clock, recalling words, and identifying patterns [21]. A higher MoCA score indicates better cognitive function, with a maximum score of 30. Typically, a score of 26 or higher is considered normal, while lower scores may suggest cognitive impairment [22].

The diagnosis of HAND was based on the Frascati criteria [23], which employs standardized neuropsychological tests (e.g., Wechsler Adult Intelligence Scale, Neuropsychological Assessment Battery) to assess cognitive function in individuals with HIV. These assessments examine the patient’s performance across a range of cognitive domains, including abstraction/executive function, attention/working memory, memory, motor skills, speed of information processing, sensory/perceptual skills, and verbal/language skills. During the diagnostic process, the patient’s scores are typically compared to age- and education-adjusted standardized scores. A diagnosis of HAND is considered if the patient exhibits scores that are at least one standard deviation (SD) below the mean in two or more cognitive domains [24]. And the neurocognitive test battery used in this study is detailed in our previous work [8]. The Frascati criteria classify HAND into three categories based on whether cognitive impairment affects daily functioning: asymptomatic neurocognitive impairment (ANI), mild neurocognitive disorder (MND), and HIV-associated dementia (HAD).

Smoking and drinking habits were assessed through self-reported questionnaires, where participants provided information on the frequency and quantity of their smoking and alcohol consumption.

Daily activity levels were evaluated using the activities of daily living (ADL) scale, which comprises 14 items across two sections. The basic ADL scale includes six items: eating, regular use of the toilet, dressing and undressing, walking, bathing, and personal grooming. The instrumental ADL scale includes eight items: using public transportation independently (i.e., knowing which bus to take and being able to travel alone), performing household chores, cooking, washing one’s clothes, making phone calls, shopping, managing personal finances, and taking medications (i.e., remembering to take medications on time and correctly). Participants or their family members rated the items based on actual performance, following the specified scoring criteria. The scale is as follows: 1 point for complete independence, 2 points for some difficulty but still able to perform the task independently, 3 points for requiring assistance, and 4 points for being unable to perform the task at all. If the participant has never performed an activity but is capable of doing so, they are rated 1 point. If they have never performed it but experience difficulty, they are rated 2 points. The minimum possible score is 14, indicating no functional impairment, while scores above 14 suggest the presence of functional impairment.

### Clinical evaluation

Demographic data were collected from all participants, including age, sex, years of education, height, weight, body mass index (BMI), and lifestyle habits. Clinical characteristics of people living with HIV (PLWH) were also documented, including CD4^+^ T cell counts, viral loads, duration of antiretroviral therapy (ART). Furthermore, scores from neurocognitive tests assessing daily function and the following seven cognitive domains were recorded.

### Biospecimen collection and processing

Blood samples were collected from participants who fasted for 12 h. Plasma samples were separated after centrifugation at 800 g for 5 min and stored at -80 °C. And Fecal samples were collected using sterile, single-use collection containers. Participants are instructed to provide a fresh sample, ensuring that it is free from contamination (e.g., urine, water, or other foreign substances), and then samples were frozen at -80 °C.

### Measurement of SCFAs

GC-MS was employed to measure the levels of SCFAs. The experimental procedure was as follows: (1) Samples were thawed on ice, and 200 µL was transferred to a 2 mL centrifuge tube. To resuspend, 50 µL of 20% phosphoric acid was added, along with 4-methylvaleric acid as an internal standard (final concentration: 500 µM). The mixture was vortexed for 2 min and centrifuged at 14,000 g for 20 min. The supernatant was transferred to an injection vial for GC-MS analysis, and 1 µL was injected with a split ratio of 10:1. (2) Separation was performed on an Agilent DB-FFAP capillary column (30 m × 250 μm × 0.25 μm). (3) Mass spectrometry analysis utilized an Agilent 5977B MSD. (4) Chromatographic peak areas and retention times were analyzed using MSD ChemStation software, and standard curves were generated to calculate SCFA concentrations.

### Measurement of gut microbiota

Metagenomic analysis was performed to evaluate the abundance of gut microbiota. The methodology used for metagenomic analysis in this study followed the procedures detailed in our previous work [8].

### Cytokine profiling

The expression of cytokine in plasma of participants was measured using Human XL Cytokine Luminex Performance Panel Premix (R&D Systems, Bio-Techne, U.S.) based on the Luminex^®^ xMAP^®^ technology. Briefly, all reagents were prepared according to the manufacturer’s instructions. A total of 50 µL of either standard, control, or sample was added to each well, followed by the addition of 50 µL of diluted Microparticle Cocktail. The plate was incubated for 2 h at room temperature (RT) on a shaker set at 800 rpm. After three washes, 50 µL of diluted Biotin-Antibody Cocktail was added to each well and incubated for 1 h at RT on the shaker. Following another three washes, 50 µL of diluted Streptavidin-PE was added, with a subsequent incubation for 30 min at RT. The plate was then read within 90 min using the Luminex^®^ 200™ system equipped with HTS and FLEXMAP 3D^®^ software. Standard curves for each analyte were generated by fitting the data to a five-parameter logistic (5-PL) curve using the software.

### Statistics

Statistical analyses were conducted using IBM SPSS Statistics (version 26.0) and GraphPad Prism (version 8.0). For continuous variables, descriptive statistics including mean and SD were calculated, while categorical variables were presented as frequencies and percentages. Group comparisons were performed using t-tests for two groups and one-way ANOVA or the Kruskal-Wallis test for three or more groups, with statistical significance defined as *p* < 0.05. *p*-values were adjusted using the Bonferroni method where appropriate. Spearman correlation was used to assess the relationships between SCFAs, gut microbiota, cognitive function, inflammation, and immune responses. All statistical tests were two-tailed unless otherwise noted.

## Results

### Participants

A total of 83 participants were included in the study: 28 in the HAND group, 28 in the HIV Control group, and 27 in the HC group. The demographic and clinical characteristics of the three groups are presented in Table 1. No significant differences were found across the groups in terms of age, gender, smoking and drinking habits, or daily activity levels. Additionally, the HAND and HIV Control groups showed no statistically significant differences in ART duration or CD4^+^ T cell counts. However, the BMI of the HC group was significantly higher than that of the PLWH groups. Notably, MoCA scores were significantly lower in the HAND group compared to both the HIV control and HC groups. Cognitive testing revealed that the HIV Control group performed better than the HAND group across all seven cognitive domains, with statistically significant differences (*p* < 0.05) observed in all domains except for Abstraction/Executive Function.

**Table 1:** The characteristics of participants

Characteristics	HC (*n* = 27)	HIV Control (*n* = 28)	HAND (*n* = 28)	*p* value
Age (years), mean ± SD	41.7 ± 10.6	39.0 ± 9.4	45.8 ± 11.5	0.058
Male, n (%)	21 (77.8)	26 (92.9)	23 (82.1)	0.284
BMI (kg/m^2^), mean ± SD	26.2 ± 4.0	23.8 ± 3.3	23.8 ± 3.2	0.020
Smoking status, n (%)	8 (29.6)	12 (42.9)	9 (32.1)	0.548
Drinking status, n (%)	13 (48.1)	17 (60.7)	10 (35.7)	0.173
ART years, mean ± SD	-	8.0 ± 5.2	9.8 ± 6.2	0.248
CD4 ^+^ T cell counts (cells/µL), mean ± SD	-	689 ± 314	887 ± 464	0.106
Viral load (TND), n (%)	-	28 (100)	28 (100)	-
Activity of Daily Living Scale, mean ± SD	14.0 ± 0.0	14.0 ± 0.0	14.1 ± 0.8	0.378
MoCA score, mean ± SD	24.7 ± 2.7	26.1 ± 2.4	21.6 ± 4.2	< 0.0001
Attention/Working Memory, mean ± SD	-	0.0 ± 0.7	−0.9 ± 0.8	< 0.0001
Abstraction/Executive Function, mean ± SD	-	0.4 ± 0.6	0.7 ± 1.0	0.109
Memory, mean ± SD	-	−0.2 ± 0.6	−1.3 ± 0.9	< 0.0001
Speed of Information Processing, mean ± SD	-	0.1 ± 0.7	1.1 ± 0.9	< 0.0001
Sensory/Perceptual Skills, mean ± SD	-	0.1 ± 0.5	−0.5 ± 0.7	< 0.0001
Verbal/Language Skills, mean ± SD	-	0.0 ± 0.9	−0.6 ± 0.9	0.009
Motor Skills, mean ± SD	-	0.1 ± 0.7	0.9 ± 1.1	0.001

### The levels of SCFAs decrease in HAND

**Fig. 1:**
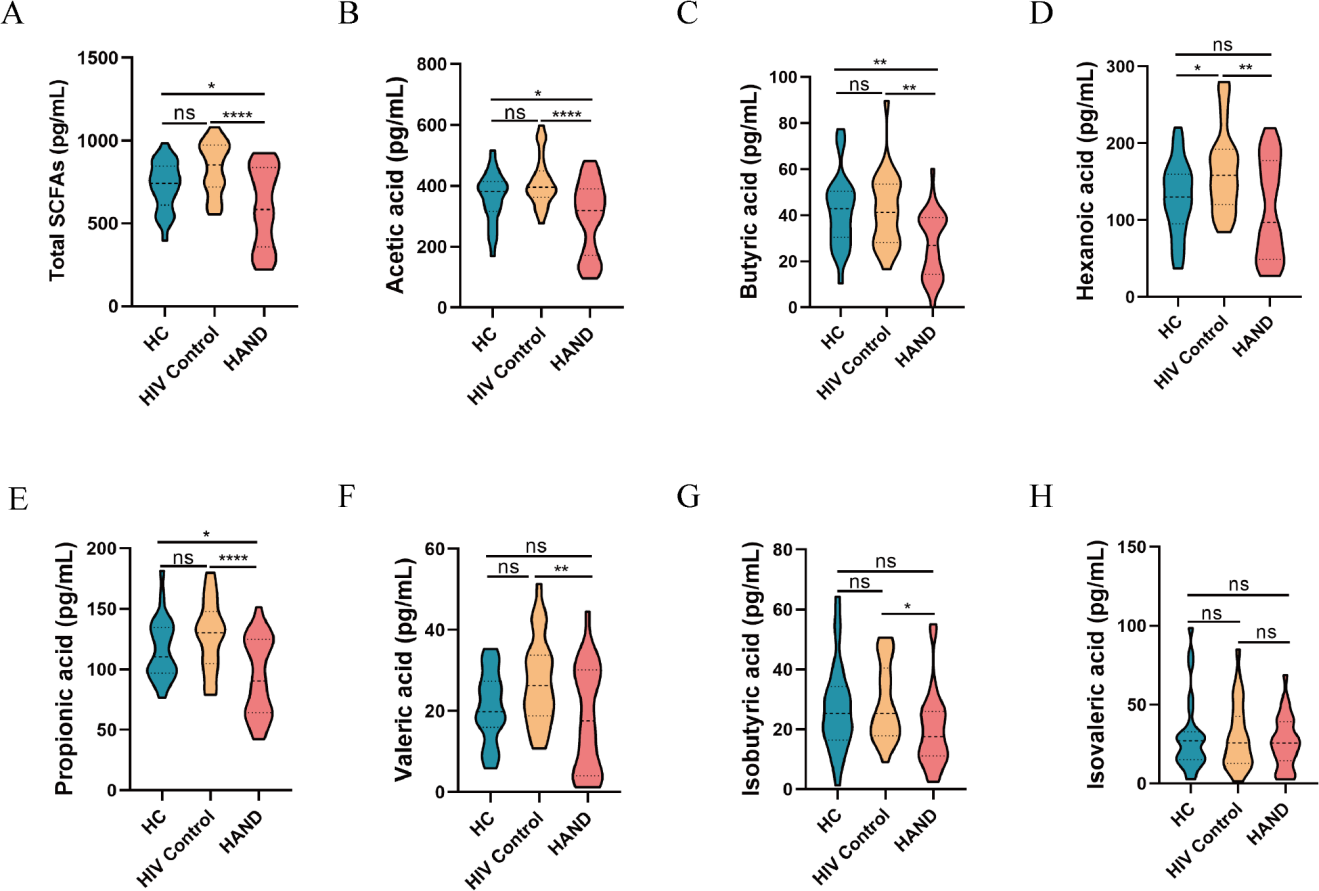
Decreased levels of SCFAs in HAND. Plasma concentrations of SCFAs were compared among HAND patients, HIV controls, and healthy controls. Statistical significance was determined using one-way ANOVA, with the following significance thresholds: ^*^p < 0.05, ^**^p < 0.01, ^***^p < 0.001, and ^****^p < 0.0001

To investigate the relationship between SCFAs and HAND, we measured plasma concentrations of acetic acid, butyric acid, hexanoic acid, propionic acid, valeric acid, isobutyric acid, and isovaleric acid in HC, HIV Control, and HAND groups. Our analysis indicated that total SCFAs, as well as specific SCFAs such as acetic acid, butyric acid, hexanoic acid, propionic acid, and valeric acid, exhibited trends toward elevated levels in the HIV control group compared to the HC group, however, most of these differences did not reach statistical significance. In contrast, these SCFAs were markedly reduced in the HAND group, which exhibited even lower levels than the HC group (Fig. 1A-F). Additionally, isobutyric acid levels were higher in the HIV control group compared to the HAND group (Fig. 1G), while isovaleric acid levels did not differ significantly among the three groups (Fig. 1H). We further analyzed SCFAs levels across HAND subtypes (ANI, MND, and HAD) and observed a downward trend in specific SCFAs, including butyric acid, acetic acid, hexanoic acid, and valeric acid, with increasing HAND severity; however, this trend lacked statistical significance (Fig. S1).

### The SCFAs-producing gut bacteria decrease in HAND

**Fig. 2:**
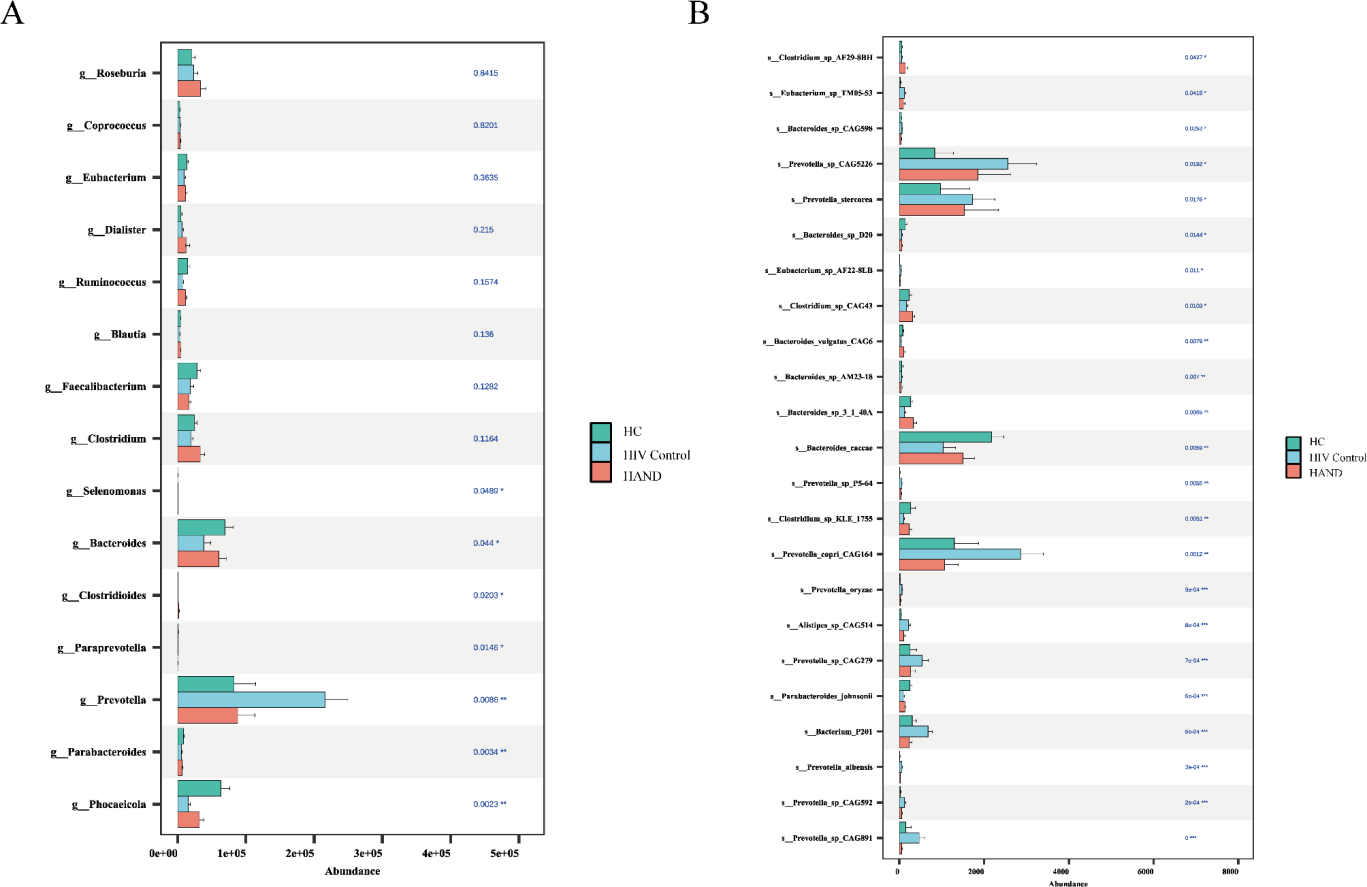
Reduced SCFA-producing gut bacteria in HAND. The Kruskal-Wallis rank sum test was used to analyze the abundance of key gut microbiota at the genus level (**A**) and species level (**B**). The x-axis represents the abundance of the microbiota. Statistical significance was denoted as ^*^p < 0.05, ^**^p < 0.01, ^***^p < 0.001, and ^****^p < 0.0001

We further investigated whether changes in SCFA-producing gut bacteria were correlated with variations in SCFA levels by performing metagenomic sequencing of fecal samples from the subjects. The Kruskal-Wallis test was performed to compare SCFAs-producing gut bacteria across the three groups. At the genus level, we observed a decrease in *Bacteroides* among PLWH compared to the HC group, while *Bacteroides* levels were higher in the HAND group than in the HIV Control group. Both *Phocaeicola* and *Parabacteroides* showed reductions in the HAND and HIV Control groups. Conversely, *Prevotella* was elevated in the HIV control group compared to the HC group but decreased in the HAND group (Fig. 2A). At the species level, the *Prevotella* species, including *Prevotella copri CAG164*, *Prevotella sp.*, and *Prevotella stercorea*, were significantly elevated in HIV Control group compared to HC group, while the HAND group exhibited the lowest levels of these species among the three groups (Fig. 2B).

### Plasma SCFAs correlate with inflammatory cytokines and cognitive function

**Fig. 3:**
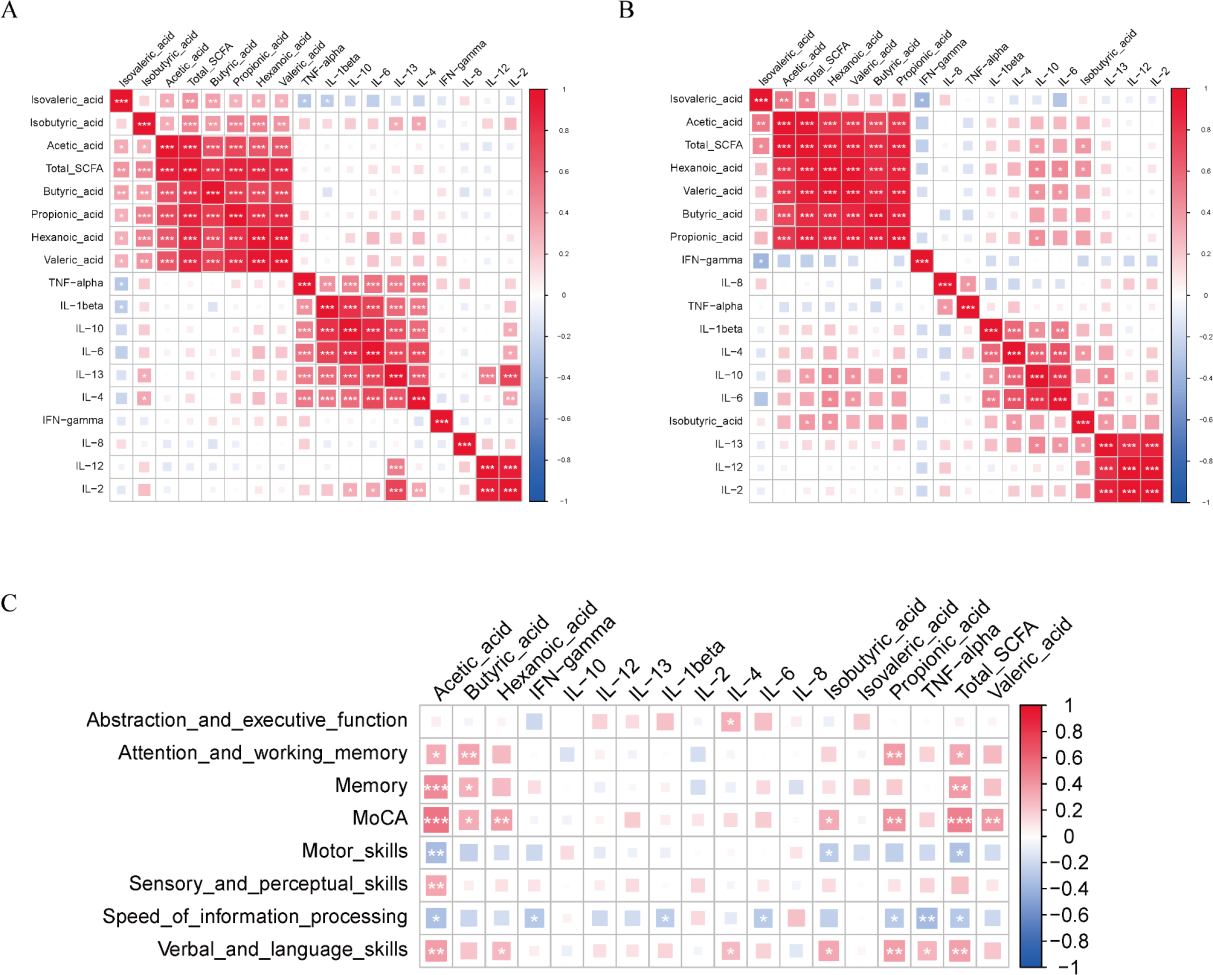
Plasma SCFAs correlate with inflammatory cytokines and cognitive function. Spearman correlation analysis was used to examine the relationship between SCFAs and inflammatory cytokines in all PLWH (**A**) and specifically in the HAND group (**B**). Further analysis examined correlations between SCFAs, inflammatory markers, and cognitive domain scores in HIV controls and HAND groups (**C**). The intensity and size of the squares indicate the strength of the correlation, ranging from negative (blue) to positive (red). Significance levels were denoted by ^*^p < 0.05, ^**^p < 0.01, ^***^p < 0.001, and ^****^p < 0.0001

SCFAs can modulate inflammation by regulating several leukocyte functions, including cytokine production [25]. To explore the association between SCFAs and inflammatory cytokines, we measured inflammatory cytokine levels in plasma. The expression levels of cytokines in the three groups are shown in Fig. S2, we found that the pro-inflammatory cytokine IL-8 was elevated in the HAND group, while several anti-inflammatory cytokines, such as IL-10, IL-4, and IL-13, were reduced in the HAND group. Correlation analysis between SCFAs and these cytokines was performed using Spearman’s method. A significant correlation was observed between SCFAs and cytokines in both the overall PLWH group and specifically the HAND group. In the PLWH group, isobutyric acid showed a positive correlation with cytokines, including IL-13 and IL-4, while isovaleric acid demonstrated an inverse relationship with cytokines, especially IL-1β and TNF-α (Fig. 3A). In the HAND group, several cytokines, such as IL-6, IL-4, IL-13, and IL-10, positively correlated with most SCFAs, whereas cytokines such as IFN-γ, TNF-α, and IL-8 showed an inverse relationship with SCFAs, though the correlations lacked statistical significance (Fig. 3B). Additionally, Spearman’s rank correlation analysis was performed between cognitive domains and SCFAs, as well as cytokines, indicating that abstraction/executive function, memory, and MoCA scores positively correlated with SCFAs, while attention/executive function was negatively correlated with cytokines, particularly IL-10 (Fig. 3C).

### Predictive value of SCFAs for HAND status and severity

**Fig. 4:**
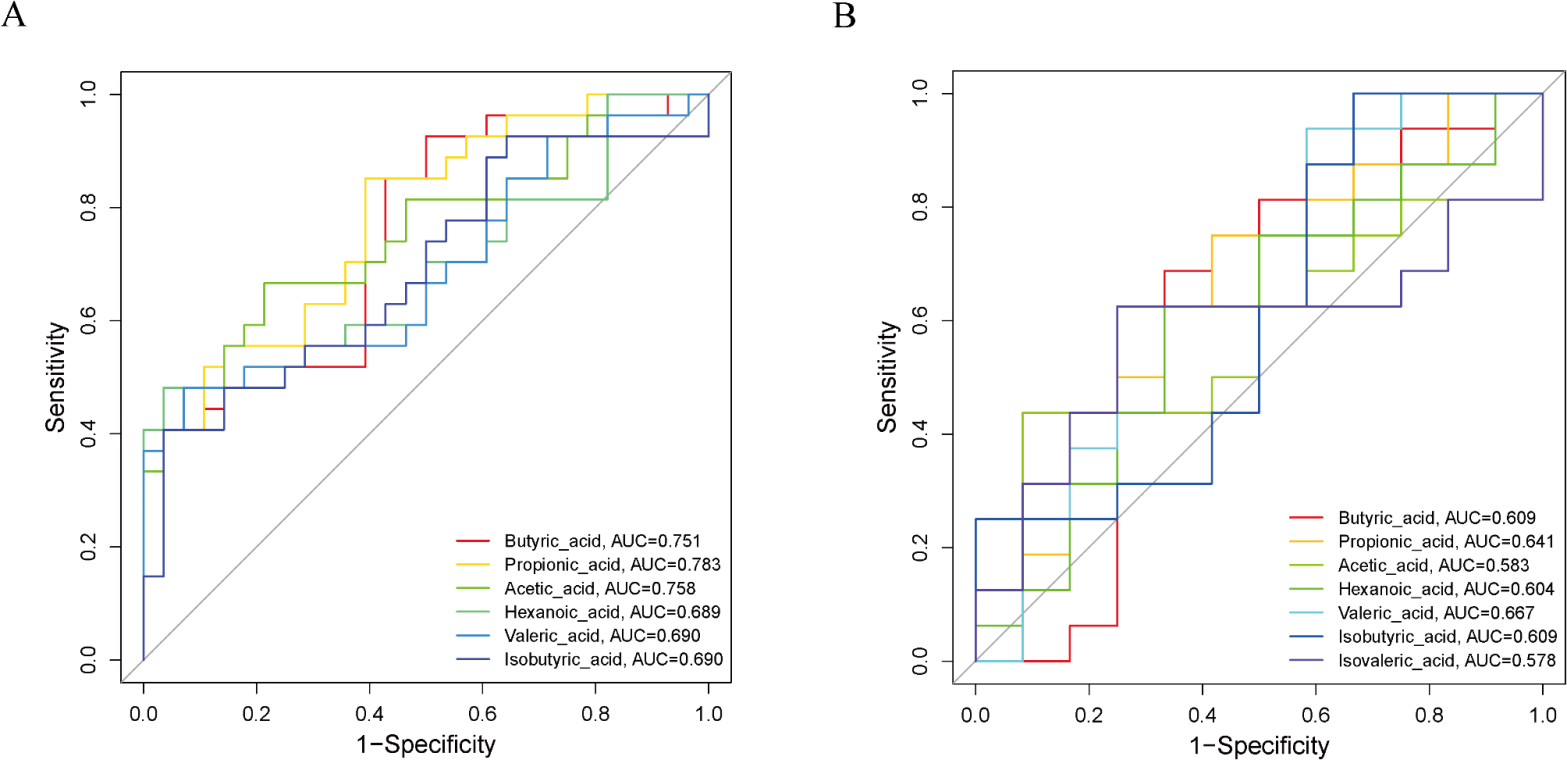
Predictive role of SCFAs in HAND status and severity. Receiver-operating characteristic (ROC) curves were generated for the differential SCFAs between HAND and HIV controls (**A**) and across different HAND subtypes (**B**). The area under the curve (AUC) was used to evaluate predictive accuracy

To assess the potential of each SCFA in determining HAND status and severity, receiver operating characteristic (ROC) curves were generated. For HAND status, most SCFAs that significantly differed between the HIV Control and HAND groups had an area under the curve (AUC) exceeding 0.70, indicating strong predictive accuracy (Fig. 4A). Notably, acetic acid (AUC = 0.758), butyric acid (AUC = 0.751), and propionic acid (AUC = 0.783) demonstrated particularly robust performance. Furthermore, HAND participants were stratified into ANI and MND/HAD subgroups, and ROC curves were employed to evaluate the predictive capacity of seven SCFAs for HAND severity. The results showed that all seven SCFAs had AUC values below 0.70, indicating limited predictive accuracy for assessing HAND severity (Fig. 4B).

## Discussion

In this study, we quantified plasma SCFAs using GC-MS and evaluated gut microbiota through fecal metagenomic sequencing. Additionally, we assessed inflammatory cytokine expression. Our findings revealed that PLWH with HAND exhibited reduced plasma SCFA levels and diminished abundance of SCFA-producing gut bacteria. Furthermore, SCFA levels were correlated with several inflammatory cytokines. These findings suggest that SCFAs may contribute to HAND pathogenesis by influencing neuroinflammation.

We investigation into plasma SCFA levels in HAND patients, revealing a reduction in SCFA levels compared to HC and HIV controls. These results align with previously reported trends of SCFAs in other neurodegenerative diseases [16, 19, 26–28]. Butyrate, a key microbial-derived SCFA, has been widely recognized for its dual role in modulating host metabolism and regulating inflammation. It serves as a major energy source for colonocytes and exhibits potent anti-inflammatory effects by inhibiting histone deacetylases (HDACs) and activating G-protein-coupled receptors (e.g., GPR41 and GPR43) [29, 30]. These pathways are implicated in the regulation of immune cell function, including the suppression of pro-inflammatory cytokine production and the promotion of regulatory T cell differentiation [31]. In the context of HAND, reduced butyrate levels may exacerbate neuroinflammation through heightened activation of microglia and peripheral immune cells, contributing to the observed cognitive decline. While our analysis demonstrated a downward trend in butyrate levels with increasing HAND severity, the lack of statistical significance warrants further investigation with larger cohorts to validate this association. This highlights the potential of butyrate as both a biomarker and a therapeutic target for HAND, deserving more in-depth exploration in future studies.

SCFAs are primarily produced by gut bacteria through the fermentation of non-digestible carbohydrates, such as dietary fiber [32, 33]. The gut microbiota responsible for producing SCFAs consists of various bacterial groups, with *Firmicutes* and *Bacteroidetes* being the dominant phyla. Key SCFA-producing species include *Faecalibacterium prausnitzii* and *Roseburia intestinalis*, which are major butyrate producers; *Bifidobacteria* species, such as *Bifidobacterium adolescentis* and *Bifidobacterium longum*, primarily generate acetate and lactate; *Akkermansia muciniphila* also contributes to SCFA production, mainly acetate, and plays a vital role in gut health [34–36]. In our study, we observed an increased abundance of SCFA-producing bacteria, including *Prevotella copri CAG164*, *Prevotella sp.*, and *Prevotella stercorea*, in the HIV control group compared to HC. However, these bacteria were reduced in the HAND group, which correlates with alterations in SCFAs, suggesting that these bacteria may be involved in SCFAs production. *Prevotella*-related species are known to produce acetate and valerate [37, 38]. These findings suggest that gut microbiota-derived SCFAs may contribute to HAND, although further research is needed to uncover the underlying mechanisms.

Accumulating evidence suggests that SCFAs can attenuate neuroinflammation in various neurodegenerative diseases through different mechanisms [39–41]. In our study, we explored the correlation between SCFAs and inflammation, finding that anti-inflammatory cytokines such as IL-10, IL-13, and IL-4 were strongly associated with SCFAs, while pro-inflammatory cytokines like IFN-γ and TNF-α showed negative correlations. Additionally, we demonstrated that changes in SCFA levels positively correlated with memory performance and MoCA scores, indicating that reduced SCFA levels negatively impacted cognition in PLWH with HAND. This relationship between inflammatory cytokines, cognition, and SCFAs mirrors findings in PD [16]. Collectively, these results suggest that diminished SCFA levels may contribute to the pathogenesis of HAND by modulating inflammation. However, plasma cytokine analysis reveals a lack of significantly elevated inflammatory markers in the HAND group, a finding that warrants further consideration. One potential explanation for this discrepancy is that neuroinflammation, a key driver in HAND pathogenesis, may not always align directly with peripheral inflammatory responses. It is plausible that plasma cytokine levels fail to fully capture the extent of neuroinflammation within the central nervous system. Given that neuroinflammatory processes may be localized to the brain, they might not be reflected in peripheral circulation due to the restrictive nature of the BBB, which impedes the passage of inflammatory mediators into the bloodstream.

Metabolomics has become a valuable tool for identifying potential biomarkers in neurodegenerative diseases. For instance, *Burté et al.* employed metabolomics to assess nine fatty acid oxidation metabolites, demonstrating high predictive accuracy for early PD and mild cognitive impairment [42]. Additionally, metabolites have been shown to enhance the AUC of models used for classifying and predicting AD [43]. Despite the growing need, effective methods for the early diagnosis and identification of HAND remain limited. Given the distinct roles of SCFAs in HAND, we hypothesized that SCFAs could serve as potential biomarkers for predicting HAND. ROC curve analysis from our study revealed that six SCFAs had predictive power for HAND onset. However, their ability to predict HAND severity was limited, likely due to the small sample size. This is consistent with previous research showing that gut and plasma SCFAs can serve as early diagnostic biomarkers, distinguishing patients with mild cognitive impairments from healthy controls, and differentiating multiple system atrophy from PD [44, 45]. Taken together, these findings suggest that SCFAs may contribute to HAND through immune modulation, and SCFAs could serve as potential biomarkers for predicting HAND.

Our study has several limitations. Frist, the limited sample size restricts the generalizability of our findings. Future studies should involve larger cohorts to enhance the applicability of these results. Second, we did not assess fecal SCFAs, which limits our ability to establish a direct link between gut microbiota and SCFA levels. Third, the cross-sectional design of our study prevents us from capturing dynamic changes in SCFAs or determining their causal relationship with HAND progression. Longitudinal studies that monitor both plasma and fecal SCFAs across different stages of HAND are needed. Additionally, the interaction between SCFAs and inflammatory responses requires further exploration, as our current understanding lacks in-depth mechanistic insights. Future research should incorporate cell and animal models to investigate how SCFAs influence HAND through immune modulation. Despite these limitations, this study highlights the potential role of SCFAs in HAND pathogenesis.

## Conclusion

Our findings suggest that plasma SCFAs may play a role in the development of HAND by modulating inflammatory responses and could potentially serve as biomarkers for predicting HAND. In individuals with HAND, plasma SCFAs and SCFA-producing gut microbiota were reduced. These decreased SCFA levels were positively correlated with pro-inflammatory cytokines and cognitive impairment, while showing a negative correlation with anti-inflammatory cytokines. Our study identifies new targets for predicting and treating HAND, underscoring the need for further research to explore the complex mechanisms by which SCFAs contribute to this condition.

## Electronic supplementary material

Below is the link to the electronic supplementary material.

Supplementary Material 1

## Data Availability

No datasets were generated or analysed during the current study.

## Abbreviations

SCFAs: Short Chain fatty acids
HIV: Human immunodeficiency virus
HAND: HIV-associated neurocognitive disorder
HC: Healthy controls
ROC: Receiver operating characteristic
AD: Alzheimer’s disease
PD: Parkinson’s disease
CNS: Central nervous system
BBB: Blood-brain barrier
LPS: Lipopolysaccharide
GC-MS: Gas chromatography-mass spectrometry
MoCA: Montreal cognitive assessment
SD: Standard deviation
ANI: Asymptomatic neurocognitive impairment
MND: Mild neurocognitive disorder
HAD: HIV-associated dementia
ADL: Activities of daily living
BMI: Body mass index
PLWH: People living with HIV
ART: Antiretroviral therapy
AUC: Area under the curve
HDACs: Histone deacetylases
GPR: G-protein-coupled receptors
