# Supplementary material for: Association between plasma short-chain fatty acids and inflammation in human immunodeficiency virus-associated neurocognitive disorder: a pilot study
Source: Lipids Health Dis. 2025 Feb 21;24:66. doi: 10.1186/s12944-025-02477-x (PMC11846350; doi:10.1186/s12944-025-02477-x)
Supplement: Supplementary file 1 — Supplementary Material 1 [file 12944_2025_2477_MOESM1_ESM.docx]

**Supplementary Figures:**

**Fig. S1:**


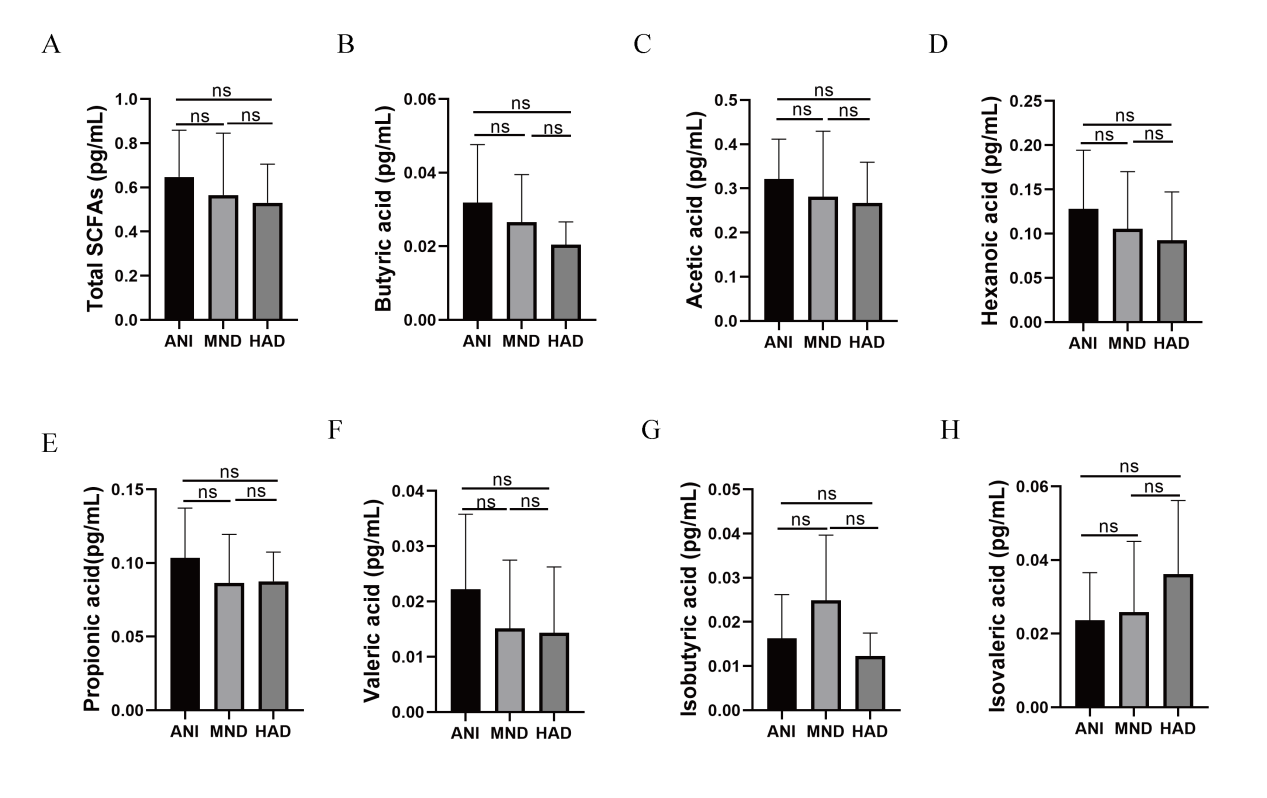


**Fig. S1. The expression of SCFAs among the three HAND subtypes (ANI, MND, and HAD).**

**Fig. S2:**


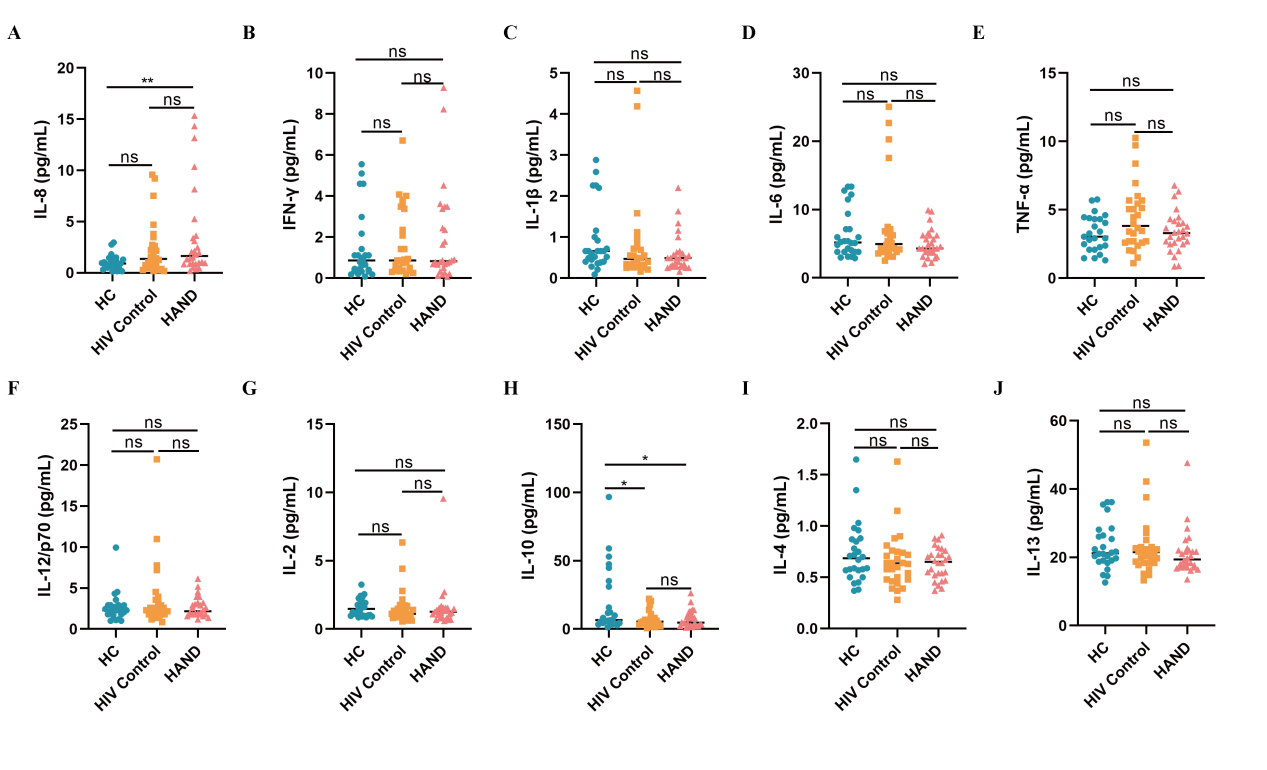


**Fig. S2 Comparisons of levels of inflammatory among HC, HIV Control, and HAND groups**

Individual values showed median with range for IL-8 (A), IFN-γ (B), IL-1β (C), IL-6 (D), TNF-α (E), IL-12/p70 (F), IL-2 (G), IL-10 (H), IL-4 (I), and IL-13 (J). ^*^*p* < 0.05, ^**^*p* < 0.01.
